# Correction: Analysis of clinicopathological factors associate with the visibility of early gastric cancer in endoscopic examination and usefulness of linked color imaging: A multicenter prospective study

**DOI:** 10.1371/journal.pone.0322354

**Published:** 2025-04-08

**Authors:** Kensuke Fukuda, Kazuhiro Mizukami, Daisuke Yamaguchi, Yuichiro Tanaka, Kazutoshi Hashiguchi, Takashi Akutagawa, Ryo Shimoda, Sho Suzuki, Tadashi Miike, Yorinobu Sumida, Hidehito Maeda, Fumisato Sasaki, Ryosuke Gushima, Hideaki Miyamoto, Keiichi Hashiguchi, Naoyuki Yamaguchi, Tetsuya Ohira, Tetsu Kinjo, Ken Ohnita, Tomohiko Moriyama, Kensei Ohtsu, Akira Aso, Ryo Ogawa, Tetsuya Ueo, Masahide Fukuda

The third author’s name is spelled incorrectly. The correct name is: Daisuke Yamaguchi. The correct citation is: Fukuda K, Mizukami K, Yamaguchi D, Tanaka Y, Hashiguchi K, Akutagawa T, et al. (2024) Analysis of clinicopathological factors associate with the visibility of early gastric cancer in endoscopic examination and usefulness of linked color imaging: A multicenter prospective study. PLoS ONE 19(11): e0312385. https://doi.org/10.1371/journal.pone.0312385
