# Supplementary figures and images for: Analysis of clinicopathological factors associate with the visibility of early gastric cancer in endoscopic examination and usefulness of linked color imaging: A multicenter prospective study
Source: PLoS One. 2024 Nov 5;19(11):e0312385. doi: 10.1371/journal.pone.0312385 (PMC11537390; doi:10.1371/journal.pone.0312385)

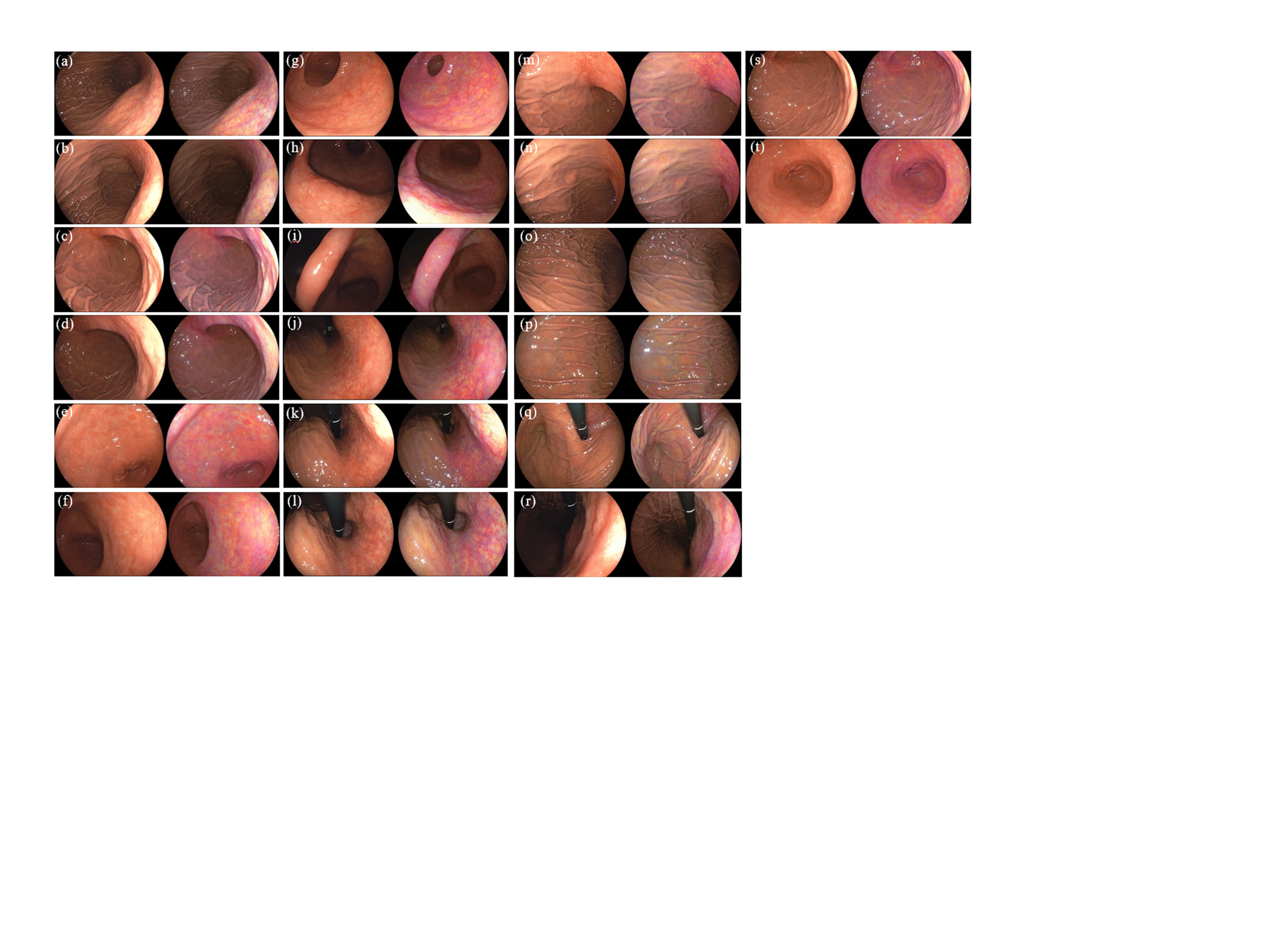

Supplement: S1 Fig — Endoscopic images was obtained for each WLI and LCI in upper, middle, lower, posterior wall of gastric body (a-c), posterior wall of upper gastric angle (d), four-quadrant views of gastric antrum (e-h), lesser curvature of gastric angle (i), lesser curvature of lower, middle, upper gastric body (j-l), anterior wall and greater curvature of lower, middle, upper gastric body(m-p), retroflexed view of cardia, posterior wall of lower gastric body (q,r), antegrade view of posterior wall of gastric angle, prepylorus (s,t). These images were not focused on the target lesion and at least one of the 20 images should include the lesion. (TIF) [file pone.0312385.s001.tif]
